# Relationship between personality traits and postpartum depression in Pakistani fathers

**DOI:** 10.1371/journal.pone.0303474

**Published:** 2024-05-14

**Authors:** Najam ul Hasan Abbasi, Ahmad Bilal, Khair Muhammad, Saba Riaz, Shakeela Altaf

**Affiliations:** 1 Department of Academic Sciences, Mianyang Normal University, Mianyang, Sichuan, China; 2 Department of Applied Psychology, The Islamia University of Bahawalpur, Bahawalpur, Pakistan; 3 Department of Psychology, Government Degree College, Balakot, Mansehra, KPK, Pakistan; 4 Department of Allied Health Sciences, Iqra University, Islamabad, Pakistan; University of Rome La Sapienza: Universita degli Studi di Roma La Sapienza, ITALY

## Abstract

The previous studies have found an association between Big Five personality traits and postpartum depression in women. The present study aimed to find out an association between Big Five personality traits and postpartum depression in a sample of Pakistani fathers. A total of 400 Pakistani fathers who had birth of a child in the past 1 month to 1 year period and had been living with their married partners were recruited purposively by using Google Form based survey from the major cities of Pakistan. The Urdu translated versions of Big Five Personality Inventory (BFI) and Edinburgh Postnatal Depression Scale (EPDS) were used as the main outcome measures to assess the relationship between personality traits and postpartum depression. The results found a significant negative and moderate association between Big Five personality traits and paternal postpartum depression except openness which had a weak association and neuroticism which had a positive and moderate association with PPPD (*r*(398) = .45). The multiple linear regression analysis found that Big Five personality traits significantly predicted paternal postpartum depression (*F*(5, 394) = 53.33, *p* = .001) except openness (B = .007, *p* = .98). The analysis of variance (ANOVA) found significant differences in paternal postpartum depression for age of father (*F*(2, 397) = 6.65, *p* = .001, ηp^2^ = .03), spouse age (*F*(2, 393) = 5.97, *p* = .003, ηp^2^ = .02), employment type (*F*(2, 395) = 9.69, *p* = .001, ηp^2^ = .04) and time spent at home (*F*(2, 397) = 6.23, *p* = .002, ηp^2^ = .03) while there were found no significant differences for education (*F*(2, 397) = 1.29, *p* = .27, ηp^2^ = .006), marital duration (*F*(2, 397) = 2.17, *p* = .11, ηp^2^ = .01), and birth number of recent child (*F*(2, 397) = 1.48, *p* = .22, ηp^2^ = .007). The study concluded that Big Five personality traits are significantly correlated with and predict paternal postpartum depression except openness which did not predict paternal postpartum depression. The occurrence of paternal postpartum depression varied significantly for age of father, age of spouse, type of employment and time spent at home.

## Introduction

Although, there is a multitude of studies available on the maternal postpartum depression (MPPD), yet there is scant research available on paternal postpartum depression (PPPD) [1]. The high prevalence rate and consequent effects on fathers’ mental health warrant further exploration of determinants of PPPD [2]. Recently, in a study of Pakistani fathers of age (20–50 years) who had a newborn of age 6 months to 1 year reported that 25.8% experienced mild postpartum depression (PPD) and 2.5% experienced severe PPD [3]. Despite its high prevalence in Pakistani society, there is scant research available on the correlates of PPPD [3].

Moreover, several demographic factors had been implicated in previous studies like education level, socioeconomic status [4] and employment status [5]. An Iranian study with 400 fathers of children aged six weeks to 1 year reported a negative relationship of education, and socioeconomic status with PPD [6].

Although, the American Psychiatric Association defines PPD in their Diagnostic and Statistical Manual, 5^th^ edition (DSM-5) as major depressive episode (with peripartum onset) occurring during pregnancy or upto 4 weeks after parturition [7], still there is no universally accepted definition of PPD [8]. The literature suggested certain gender differences in occurrence of PPD such as indecisiveness, irritability, and restricted range of emotions which are more prominent in men [9]. So, PPPD is defined as the experience of moderate to severe depression diagnosed shortly after or upto one year following the birth of a child [8, 10]. Previous studies reported a prevalence of 4–25% of PPD in fathers [11, 12]. Fathers are more likely to experience PPD in the first 3–6 months postpartum period [13]. The partners of mothers who suffer from postpartum depression (PPD) are at increased risk for developing PPPD. The previous studies reported that 24% to 50% of men suffered from PPD whose partners suffered from PPD [14]. When the mothers experienced moderate to severe depression, the chances of development of PPD in their husbands rose to over 40% [15]. The partners of women experiencing PPD experience less support, and more confusion, anger, frustration and uncertainty [16]. The men with previous history of depression or younger age are more likely to develop depression in the postpartum period. On the other hand, cultural stigma prevents men to report and seek help for their depression in the postpartum period [17].

The previous literature has found a significant positive correlation between personality traits like neuroticism and postpartum depression [18]. But this association has only been tested for mothers. A few studies explored relationship between other Big Five personality traits and postpartum depression such as extraversion, agreeableness, openness, and conscientiousness. These other personality traits were found to be negatively correlated with postpartum depression in women [19, 20]. The study by (Peñacoba-Puente et al., 2016) also found that one personality trait, openness did not predict postpartum depression [20]. The studies exploring the mechanisms involved in connection between personality traits and postpartum depression found that high amount of rumination and worries are exhibited by persons showing neuroticism which in turn linked with higher occurrence of depressive symptoms [21]. Another study reported that high levels of self efficacy mediated the relationship between personality traits (extraversion, agreeableness, conscientiousness) and postpartum depression [22].

The PPD not only affects one’s psychological health rather it significantly disturbs one’s relationship with partners and children and results in marital difficulties and lowered communication with the family members [23]. PPPD is found to be associated with increased levels of frustration and isolation in fathers which impact paternal involvement with children [24]. Moreover, the postnatal mental health of fathers plays an important role in the cognitive development of children [25]. Hence, the screening and management of fathers for PPD at the maternity clinics is warranted [26, 27] so that PPPD can be managed in a timely manner. Therefore, the present study was designed with an aim to find out the relationship between Big Five personality traits and PPD in Pakistani fathers. The study also aimed to predict PPD from Big Five personality traits and to find out the demographics of PPD in Pakistani fathers.

## Materials and methods

### Study design

The current study employed the cross sectional and survey research design. The study period was December 2020 through April 2021.

### Participants

A total of 428 married men from the major cities of Pakistan participated in the online survey. The responses of 400 married men who were currently living with their married partners and had a birth of a child in the past year were included in the study and final analysis. The age of the participating men were 18–45 years (*M* = 28.50, *SD* = 5.94). All the participants were enrolled after signing the written informed consent.

#### Inclusion criteria

The men of age 18–45 years diagnosed with postpartum depression who were currently living with their married partners and had the birth of their baby within the 1 month to 1 year period were included in the study.

#### Exclusion criteria

The men who had not been diagnosed with postpartum depression or those currently not living with their married partners were excluded from the study. The men who were suffering from any medical or psychiatric illness other than PPPD were also excluded from the study.

### Measures

#### Demographic information & informed consent form (DI&ICF)

All the participating men were required to fill the essential informed consent form prior to participate in the study. The DI&ICF contained information about the age of participating men, age of spouse, education, type of employment, marital duration, birth number of recent child and number of hours spent at home each day.

#### Edinburgh Postnatal Depression Scale (EPDS)

The EPDS is a 10 items scale used to assess the occurrence of postnatal depression. The scale has 4 point likert type options. The maximum possible score on the scale is 30. A score of 10 or above warrants the possibility of postnatal depression, however, a score of 13 or above shows a possibility of postnatal depression of moderate intensity [28]. The EPDS has been found to be a legitimate tool to assess postpartum depression in men [29]. The EPDS was standardized in Urdu language by following the forward and back translation method. The Cronbach Alpha reliability of the Urdu version is .71.

#### Big Five Personality Inventory (BFI)

The BFI is a 44 items 5-point likert type scale used to assess personality characteristics on five main dimensions of personality namely extraversion, agreeableness, conscientiousness, neuroticism, and openness [30]. The 44 items ask questions related to each dimension of the human personality. The BFI was standardized in Urdu language by following the forward and back translation method. The Cronbach Alpha reliability of the Urdu version is .61.

### Procedure

The study was duly approved by the Ethics Review Board of Institute of Psychological Research Pakistan vide No. IPR/ERB/PPPD/2020/001 dated: 09-11-2020. All the participating fathers signed the written informed consent before their enrollment. A Google Form based survey containing a complete set of questionnaires including informed consent, demographic information form, and Urdu versions of both EPDS, and BFI was floated through different social media platforms and personal contacts. The Google form was used as there was smart lockdown in the country and it was difficult to recruit men directly. A sample size of 384 was computed keeping in view the adult population of married men in Pakistan based on Population Census 2017 [31] with a 95% confidence interval and 5% error of measurement. The pre selected *α* level was .05 with a power of 0.9 (90%) and an effect size of 0.1 (10%). The pre selected type II error was 0.1 (10%). These calculations corresponded well with the statistical testing methods like correlation, multiple linear regression, and one way ANOVA. The online sample size calculator was used to compute the sample size [32]. The sample size for this study was marginally inflated for increasing the accuracy of statistical analyses.

### Statistical data analyses

The Statistical Package for the Social Sciences (SPSS), version 25 (SAS Institute, Cary, NC) was used to statistically analyze the data. The demographic characteristics of the participating men were calculated through frequency distribution and percentages. One way Analysis of Variance (ANOVA) was used to compare demographic differences in PPPD. Pearson correlation analysis was used to determine if an association between PPPD and personality traits existed. Multiple linear regression analysis was used to predict PPPD from the personality traits. The effect sizes of .02, .09, and .25 were considered as the small, medium, and large effect size respectively and were denoted by partial eta square (ηp^2^) [33].

## Results

### Demographic characteristics

The Table 1 gives the demographic characteristics of the participating men.

**Table 1:** Frequency distribution of demographic variables (*N* = 400).

Demographic Variables	Characteristics	F	%
**Age (years)**	18–27	196	49.0
*M* = 28.50	28–36	155	38.8
*SD* = 5.94	37–45	49	12.3
**Spouse’s Age (years)**	16–25	255	63.7
*M* = 24.98	26–34	116	29.0
*SD* = 5.08	35–43	29	7.2
**Education**	≤ Senior High School	212	53.0
	Bachelors	167	41.75
	Masters	21	5.25
**Type of Employment**	Business	211	52.75
	Government Job	103	25.75
	Private Job	86	21.5
**Marital Duration (years)**	1–7 years	312	78.0
	8–15 years	70	17.5
	16–22 years	18	4.5
**Birth Number of Recent Child**	1^st^/2^nd^	299	74.75
	3^rd^/4th	84	21.0
	5^th^ or More	17	4.25
**Time Spent at Home (per day)**	≤ 12 Hours	42	10.5
	12 hours	194	48.5
	24 hours	164	41.0

*Note*. *M* = Mean, *SD* = Standard Deviation

#### Correlation analysis

The Table 2 describes the correlation analyses between paternal postpartum depression and personality traits. All the personality traits had a significant association with PPPD. The extraversion, agreeableness, and conscientiousness had a moderate negative association with PPPD whereas openness had a weak negative association with PPPD. On the other hand, neuroticism had moderate positive association with PPPD.

**Table 2:** Pearson’s correlation between pppd and personality traits (*N* = 400).

Variables	*M*	*SD*	1	2	3	4	5	6
PPPD	11.36	4.89	-					
Extraversion	3.21	.47	-.45**	-				
Agreeableness	3.55	.55	-.49**	.42**	-			
Conscientiousness	3.34	.46	-.46**	.42**	.46**	-		
Neuroticism	2.83	.50	.45**	-.43**	-.26**	-.38**	-	
Openness	3.20	.43	-.21**	.32**	.33**	.27**	-.08	-

**p < .01

*Note*. *M* = Mean, *SD* = Standard Deviation

#### Multiple linear regression analysis

The Table 3 gives the results of multiple linear regression analysis used to predict paternal postpartum depression from personality traits. The model was significant overall and explained 63% of the variance in paternal postpartum depression. The personality traits were found to impact the occurrence of PPPD in 63% of the cases which clearly demonstrates the strong predictive effect of personality traits on PPPD. The personality traits significantly predicted (*F*(5, 394) = 53.33, *p* = .001) 63% change in paternal postpartum depression. All the personality traits significantly predicted paternal postpartum depression except openness (B = .007, *p* = .98).

**Table 3:** Multiple regression analysis (*N* = 400).

Variables	*B*	*SE*	*β*	*t*	*p*
Extraversion	-1.62	.49	-.15	-3.27	.001
Agreeableness	-2.44	.40	-.27	-5.99	.001
Conscientiousness	-1.81	.49	-.17	-3.66	.001
Neuroticism	2.42	.43	.25	5.61	.001
Openness	.007	.48	.001	.01	.98
***R*** ^***2***^	.63				
***F*** *(df)*	53.33 (5, 394)				
***p***	.001				

DV = PPPD

*Note*. *M* = Mean, *SD* = Standard Deviation

#### Analysis of variance for demographic differences

The Table 4 outlines the results of analysis of variance (ANOVA) used to compare the differences in paternal postpartum depression across demographic variables. The analysis found significant differences in paternal postpartum depression for age of father (*F*(2, 397) = 6.65, *p* = .001, ηp^2^ = .03), spouse age (*F*(2, 393) = 5.97, *p* = .003, ηp^2^ = .02), employment type (*F*(2, 395) = 9.69, *p* = .001, ηp^2^ = .04) and time spent at home (*F*(2, 397) = 6.23, *p* = .002, ηp^2^ = .03) while there were found no significant differences in paternal postpartum depression for education (*F*(2, 397) = 1.29, *p* = .27, ηp^2^ = .006), marital duration (*F*(2, 397) = 2.17, *p* = .11, ηp^2^ = .01), and birth number of recent child (*F*(2, 397) = 1.48, *p* = .22, ηp^2^ = .007). The Least Square Difference (LSD) Post Hoc analysis revealed that younger age of father (*M* = 12.25, *SD* = 4.54), younger age of spouse (*M* = 11.93, *SD* = 4.67), having business as employment type (*M* = 12.14, *SD* = 4.53), and spending less than 12 hours at home (*M* = 13.85, *SD* = 4.93) were significantly associated with having postpartum depression.

**Table 4:** Analysis of variance (*N* = 400).

1	Age (years)	18–27 (n = 196)	28–36 (n = 155)	37–45 (n = 49)	F(2, 397)	*p*	ηp^2^
	**Variable**	***M (SD)***	***M (SD)***	***M (SD)***			
	PPPD	12.25 (4.54)	10.60 (4.99)	10.20 (5.39)	6.65	.001	.03
**2**	**Spouse’s Age (years)**	**16–25 (n = 255)**	**26–34 (n = 116)**	**35–43 (n = 29)**	**F(2, 393)**	***p***	**ηp** ^**2**^
	**Variable**	***M (SD)***	***M (SD)***	***M (SD)***			
	PPPD	11.93 (4.67)	10.51 (5.04)	9.34 (5.53)	5.97	.003	.02
**3**	**Education**	**≤ SHS (n = 212)**	**Bachelors (n = 167)**	**Masters (n = 21)**	**F(2, 397)**	***p***	**ηp** ^**2**^
	**Variable**	***M (SD)***	***M (SD)***	***M (SD)***			
	PPPD	11.62 (4.83)	11.22 (4.89)	9.90 (5.50)	1.29	.27	.006
**4**	**Employment Type**	**Business (n = 211)**	**Govt Job (n = 103)**	**Private Job (n = 86)**	**F(2, 395)**	***p***	**ηp** ^**2**^
	**Variable**	***M (SD)***	***M (SD)***	***M (SD)***			
	PPPD	12.14 (4.53)	9.62 (5.17)	11.51 (4.88)	9.69	.001	.04
**5**	**Marital Duration (years)**	**1–7 (n = 312)**	**8–15 (n = 70)**	**16–22 (n = 18)**	**F(2, 397)**	***p***	**ηp** ^**2**^
	**Variable**	***M (SD)***	***M (SD)***	***M (SD)***			
	PPPD	11.56 (4.77)	11.01 (5.14)	9.22 (5.73)	2.17	.11	.01
**6**	**Birth No of Recent Child**	**1** ^**st**^ **/2nd (n = 299)**	**3** ^**rd**^ **/4th (n = 84)**	**5**^**th**^ **or more (n = 17)**	**F(2, 397)**	***p***	**ηp** ^**2**^
	**Variable**	***M (SD)***	***M (SD)***	***M (SD)***			
	PPPD	11.12 (4.88)	12.10 (5.03)	12.00 (4.21)	1.48	.22	.007
**7**	**Time Spent at Home (per day)**	**<12 Hours (n = 42)**	**12 Hours (n = 194)**	**24 Hours (n = 164)**	**F(2, 397)**	***p***	**ηp** ^**2**^
	**Variable**	***M (SD)***	***M (SD)***	***M (SD)***			
	PPPD	13.85 (4.93)	11.08 (4.89)	11.05 (4.73)	6.23	.002	.03

*Note*. *M* = Mean, *SD* = Standard Deviation

## Discussion

Pakistan is predominantly a patriarchal society where males are not considered to express their emotions or to report symptoms of depression either [3]. The new fathers become likely to experience depression in the wake of changing relationships and roles [4]. The present study found a negative correlation between personality traits and PPPD except neuroticism which was found to be correlated positively. These findings are in accordance with the previous studies [18, 20, 34]. Similarly, the present study significantly predicted PPPD from personality traits except openness. This finding has also been reported by an earlier study [20, 34].

Interestingly, the previous literature regarding the relationship between personality traits and MPPD reported the similar findings [18–20, 34]. The neuroticism was found to be positively correlated with PPD in women. The extraversion was found to be negatively correlated with PPD in women [18, 20]. The other three personality traits including agreeableness, conscientiousness, and openness were found to be negatively correlated with PPD in women [19] whereas only one study reported contradictory findings where no significant relationship was found between agreeableness, conscientiousness, and openness and PPD in women one year after the birth of the child [20]. All other findings regarding the occurrence of PPD in women are identical to the findings of the present study. So, further studies need to explore factors other than gender differences in the relationship between personality traits and PPD [34].

The relationship between age of father and PPD is also inconclusive. Some studies reported that young age (<30 years) predicted PPD in men [35] while some other studies suggested that the older age predicted PPD in men [36]. A study with UK Millennium Cohort revealed an association between young age of fathers and PPPD [37]. The discrepancies in findings with the previous studies may occur due to differences in sample size, research design and settings [6]. The current study also found an association between young age of father and PPPD which in part be attributed to pressures of parenting. Moreover, the young age of spouse had also been found to be associated with increased incidence of PPPD in the current study. The young age of both fathers and their spouses put a great strain on the couple in terms of parenting which in turn contribute towards the development of PPPD.

The current study found no association between education of fathers and PPPD. However, a study with UK Millennium Cohort showed that lower educational levels were associated with PPPD [37]. Similarly, a study by Nishimura et al. (2015) [27] reported no association between employment status of fathers and PPD. However, Giallo et al. (2014) [38] reported that work environment predicted PPD in men. Similarly, a previous study reported an association between employment status and PPD in men [39]. The nature of employment like part time also predicted PPD in some men in previous studies [35]. Another study conducted with Ethiopian fathers reported that fathers who were not satisfied with their income were three times more likely to experience PPPD [40]. The transition to parenthood puts pressure on the fathers to provide livelihood for their family. This pressure may lead to experience of depression in general [40]. The current study showed that fathers with business or private job showed more postpartum depression than fathers having government job. This may be due to financial pressures experienced due to COVID-19 waves in the country.

Though, the previous studies showed an association between marital support and satisfaction and PPPD [41] but the current study did not reveal a significant association between marital duration and PPPD. The increased marital duration was believed to have an impact on PPPD as marital duration was associated with marital support and satisfaction but the present study did not establish this connection for Pakistani fathers.

The previous studies reported that birth of first born child was more likely to predict paternal postpartum depression [42] but the current study did not find an association between the birth number of recent child and PPPD.

The current study found differences in PPPD in fathers spending time at home. Those fathers who spent less than 12 hours at home were had more PPPD as compared to those fathers who spent 12 hours or more than 12 hours at home. However, a recent study found no difference in PPPD among fathers who spent more time at home or not [43].

### Limitations & recommendations

The present study has certain limitations which have been outlined below. The recommendations to overcome these limitations have also been given.

1. The fathers suffering from PPPD had been approached only through Google Forms. The future studies should approach and recruit fathers with PPPD directly.
2. The generalizability of findings and strength of evidence could be increased if future studies should employ comparative or experimental research designs.
3. The present study did not measure the impact of marital satisfaction on PPPD. Marital satisfaction could be an important predictor of PPPD.
4. The present study did not include the type of family system as a demographic correlate of PPPD which should be included in future studies.
5. The present study only included those fathers who were living currently with their married partners. The future studies should also include those fathers who have been living separately from their married partners.

## Conclusion

The present study concluded that personality traits significantly predicted paternal postpartum depression except openness. Further, all personality traits have a significant association with paternal postpartum depression. Neuroticism was found to be positively associated with PPPD while all other personality traits were found to be negatively associated with PPPD. Certain demographic characteristics like age of father, age of spouse, type of employment of father, and time spent at home were found to be associated with paternal postpartum depression while other demographics such as education of father, marital duration, and birth number of recent child were found to have no significant association with PPPD. It is suggested that future studies should be conducted with a diverse sample of fathers by including more demographic characteristics to warrant more generalizability.

## Implications

The screening of fathers for both personality traits and PPD at the maternity clinics is an important implication of the present study. This screening will help identify fathers with risk of developing PPD at the eve of birth of a child. The early identification of those fathers who are at risk would allow them to go for early treatment. Moreover, better support systems may be developed for those fathers who are at risk of developing PPD. Hence, early identification and treatment would yield better interaction with the newborn child, thereby, improving family functioning [44].
